# The lateral trauma position: What do we know about it and how do we use it? A cross-sectional survey of all Norwegian emergency medical services

**DOI:** 10.1186/1757-7241-19-45

**Published:** 2011-08-04

**Authors:** Sabina Fattah, Guri R Ekås, Per Kristian Hyldmo, Torben Wisborg

**Affiliations:** 1 Hammerfest Hospital, Department of Anesthesiology and Intensive Care, Finnmark Health Trust, Hammerfest Norway; 2 Norwegian Air Ambulance Foundation. Department of Research. Drøbak, Norway; 3 Kongsvinger Hospital, Innlandet Health Trust. Kongsvinger, Norway; 4 Sørlandet Hospital Health Trust, Arendal & Kristiansand, Norway; 5 University of Tromsø, Institute of Clinical Medicine, Tromsø, Norway

## Abstract

**Background:**

Trauma patients are customarily transported in the supine position to protect the spine. The Airway, Breathing, Circulation, Disability, and Exposure (ABCDE) principles clearly give priority to airways. In Norway, the lateral trauma position (LTP) was introduced in 2005. We investigated the implementation and current use of LTP in Norwegian Emergency Medical Services (EMS).

**Methods:**

All ground and air EMS bases in Norway were included. Interviews were performed with ground and air EMS supervisors. Questionnaires were distributed to ground EMS personnel.

**Results:**

Of 206 ground EMS supervisors, 201 answered; 75% reported that LTP is used. In services using LTP, written protocols were present in 67% and 73% had provided training in LTP use. Questionnaires were distributed to 3,025 ground EMS personnel. We received 1,395 (46%) valid questionnaires. LTP was known to 89% of respondents, but only 59% stated that they use it. Of the respondents using LTP, 77% reported access to written protocols. Flexing of the top knee was reported by 78%, 20% flexed the bottom knee, 81% used under head padding. Of 24 air EMS supervisors, 23 participated. LTP is used by 52% of the services, one of these has a written protocol and three arrange training.

**Conclusions:**

LTP is implemented and used in the majority of Norwegian EMS, despite little evidence as to its possible benefits and harms. How the patient is positioned in the LTP differs. More research on LTP is needed to confirm that its use is based on evidence that it is safe and effective.

## Background

The traditional method for moving and transporting trauma patients has been with the patient in a supine position on a backboard to protect the spine. These are the guidelines that are taught presently in standard traumatology courses such as the Advanced Trauma Life Support (ATLS) and Prehospital Trauma Life Support (PHTLS) courses [1-3].

In a severely injured patient, it is essential that airways are maintained to prevent hypoxia and/or hypoventilation; there is a consensus that securing airways takes first priority [1-3]. Several studies have found that hypoxia is harmful in patients with traumatic brain injury (TBI) [4-8].

TBI is a major contributor to loss of life years and function years, and emergency personnel seek to reduce secondary insults to a minimum. According to international resuscitation guidelines [9,10], the lateral-recovery position should be used when an unconscious patient is breathing to maintain airway patency; it is further stated that "efforts to protect the cervical spine must not jeopardize oxygenation and ventilation" [9]. The supine position may be dangerous for unconscious patients because of the risk of obstruction by soft tissue/tongue, and aspiration of blood, frank vomitus, or silent regurgitation of gastric content. It is theoretically possible to tip a backboard sideways when the patient is vomiting, but in practice this may not be fast enough in the back of an ambulance en route to hospital.

There are controversies about the optimal method of airway management [8,11,12] and the effect of endotracheal intubation (ETI), as well as a lack of consensus about who should perform ETI. In Norway, emergency medical technicians (EMTs) and paramedics do not perform ETI unless there is a total loss of airway reflexes, and a supraglottic device has in many services replaced the endotracheal tube (for the same indication). According to Scandinavian recommendations [13], rapid sequence intubation or ordinary ETI is not an option for paramedics or general practitioners not skilled in anesthesia.

Unstable spinal injuries may have a devastating effect on patients, and cervical spine protection by a rigid collar (c-collar) is regarded as part of airway management [1,2]. The degree of protection offered by a c-collar has been challenged [14,15]; it may even be harmful [16]. The effectiveness and possible harm by immobilization on a spine board is also debated [15].

Modifications of the standard recovery position have been tested with regard to their effect on the spine [17,18]. Based on the existing guidelines, the lack of capacity for drug assisted endotracheal intubation in Norwegian ground EMS, and the presumed safe log-roll in ATLS/PHTLS [1,2] a new method was developed. In 2005 the ground ambulance emergency medical system (EMS) of Agder in southern Norway introduced the method called the lateral trauma position (LTP).

**Figure 1:**
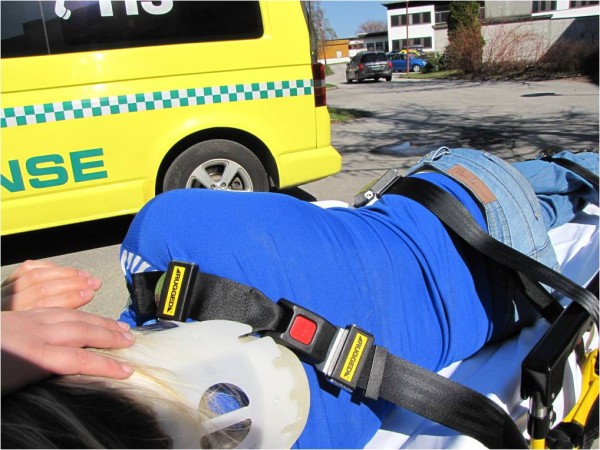
**The lateral trauma position**. A patient has been positioned in the lateral trauma position on a stretcher. Observe that the most cephalic stretcher belt has been placed above the shoulder to prevent forward movement on the stretcher.

After checking airways and breathing, the unconscious trauma patient is carefully rolled to a lateral recovery position, maintaining manual in-line stabilization with the c-collar in place (see Table 1 and Figure 1). The method was described in the EMS protocols, a teaching video was made, and training and retraining was instituted locally. PHTLS Norway has adopted LTP in their educational program (Sindre Mellesmo, personal communication). In addition to being demonstrated in PHTLS courses, LTP is now part of the written protocols in some Norwegian EMS and is described in the Scandinavian recommendations [13]. However, this implementation was never systematized [19], and nor are there clear national guidelines about how LTP is performed; e.g., with respect to which leg (top or bottom) to flex when positioning the patient.

**Table 1:** Lateral trauma position

• Check airways (look, listen, feel).
• Apply chin lift/jaw thrust, suction if needed.

• Apply stiff neck collar.

• If the patient is unresponsive, but has spontaneous respiration: Roll patient to lateral/recovery position while maintaining head/neck position.

• Roll to side that leaves the patient facing outwards in ambulance coupé.

• Transfer to ambulance stretcher (Scoop-stretcher, log-roll onto stretcher mattress, or use multiple helpers, lifting by patient's clothing).

• Support head, secure with three belts (across legs, over hip, over shoulder)

• Manual support of head, supply oxygen, observation, suction, BVM (big valve mask) ventilation when needed.

New procedures and interventions should ideally be introduced only after research has determined their efficiency and risks have been ruled out. There are obviously good arguments for the use of LTP. At the same time, minimal changes to the spinal position may be harmful to the trauma patient. The aim of this study was to investigate to what extent LTP is actually used on patients, and how it is actually performed (i.e., top or bottom leg flexed and position of head) in Norwegian EMS.

## Methods

Data were collected from three sources: ground EMS stations or regional supervisors through telephone interviews, EMS personnel through written questionnaires distributed through station supervisors, and, finally, through telephone or e-mail interviews with air EMS (helicopter and fixed wing) supervisors. Questionnaires to the ground EMS personnel were distributed continuously after approaching their supervisors. One e-mail was later sent to ground EMS supervisors asking them to remind the ground EMS personnel of the questionnaire.

The study period was from March 2010 to February 2011. During this period, there were no campaigns or official endorsements aimed at ambulance personnel regarding the positioning of trauma victims. Ethical approval from the Regional Ethics Committee was received in February 2010 (document reference 2010/107-4).

Returned questionnaires were excluded if respondents were not intended to participate (apprentices and students: 20 questionnaires), they were blank forms (two questionnaires), or there were errors (all alternatives ticked off [one questionnaire] or obviously inconsistent answers [one questionnaire]). All of the results are based on valid answers to specific questions (some questions were left blank by some respondents).

Confidence in applying the LTP method responses was categorized as "confident" or "not confident." The first comprised the alternatives "reasonably confident" and "confident," and the latter comprised "not entirely confident," "not confident," or blank answers.

## Results

**Figure 2:**
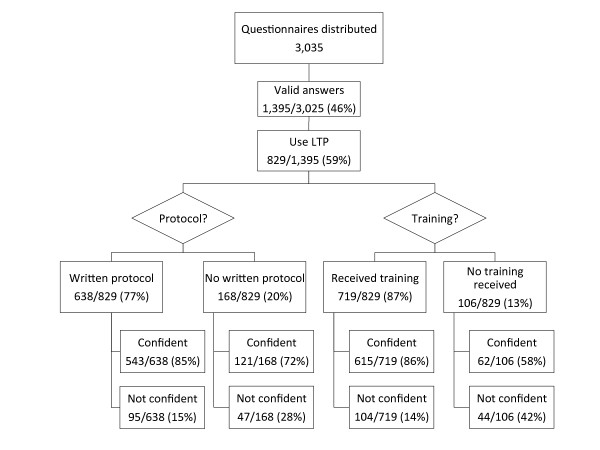
**Results of EMS personnel questionnaire**. Respondents were asked to describe whether they felt confident or not confident in the use of the lateral trauma position.

We identified 206 ground EMS supervisors (either at the station or regional level) through information provided by the Regional Health Trusts. Of these, 202 were successfully contacted and 201 agreed to participate. According to our investigations, there were 292 EMS stations in Norway at the time of the study, and ground EMS personnel from 96% (279/292) of these stations were invited to participate. Questionnaires were distributed to 3,025 ground EMS personnel. Of these, 1,395 (46%) submitted valid completed questionnaires. The results are summarized in Figure 2 and described below. Of the air EMS, 23 of the 24 medical supervisors approached by e-mail or telephone interview agreed to participate.

### Ground EMS: Implementation on a system level

The EMS supervisors reported that LTP is used in 75% (151/201) of the ground EMS. Written protocols were present in 67% (101/151) of services using LTP, and 73% (110/151) had provided training. The majority of the ground EMS supervisors were uncertain about when LTP was adopted in their service. "Not known" was reported by 51/201 and 46/201 did not answer the question. Out of the 201 responding supervisors, 26 reported that the lateral-recovery position was introduced within the last 2 y, 50 said within the last 3-5 y, 27 said within the last 10 y, and one person said that it was introduced >10 y ago.

### Ground EMS: Implementation on an individual level

LTP was familiar to 89% (1,239/1,395) of the ground EMS personnel, but only 59% (829/1,395) answered that they use it. Of those who use LTP, 87% (719/829) answered that they had received training in the use of LTP. Of these, 42% (303/719) stated that they had received training through a course; 71% (214/303) of these respondents reported that they had learned LTP at a PHTLS course. Thus, a total of 26% (214/829) of those using LTP had taken part in a PHTLS course in which LTP was taught.

Among those who reported being given training, 86% (615/719) felt confident using LTP. Of those who reported not being given training, the percentage of confident personnel was 58% (62/106). See Figure 2 for details.

Of the respondents who use LTP 77% (638/829) reported having access to written protocols. Of these, 85% (543/638) felt confident in the use of LTP, whereas 72% (121/168) of those who did not have access to a written protocol felt confident using the method.

Eighty-four percent (697/829) of those who use LTP answered a question regarding positioning of the lower extremities. The majority reported flexing the upper leg (78%; 544/697), whereas flexion of the lower leg was reported by 20% (137/697).

Positioning of the head was also investigated. Of the ground EMS personnel using LTP, 95% (785/829) answered the question about positioning of the head. Of these, 81% (633/785) reported putting padding under the head, such as a pillow or similar item. Seven percent (55/785) use a combination of padding and putting the head on the lower arm, 10% (82/785) rest the head on the lower arm alone, and less than 1% (7/785) rest the head on the ground.

### LTP implementation in the air EMS services

LTP was used by 52% (12/23) of the air EMS services, although only one service had a written protocol. Training was given in three services. Several of the fixed-wing air ambulance service supervisors commented that this is not relevant in their service, because the air EMS is primarily a transport service for stabilized patients.

## Discussion

This study shows that the majority of ground EMS use LTP. The majority of those using it also possesses written protocols and is provided with training in the use of LTP.

According to the Utstein Formula of Survival [20], medical science, educational efficacy, and local implementation are all necessary factors for survival. In the case of LTP, we have no medical science about the position that would indicate if it indeed is the optimal method for transporting unconscious trauma patients. We have little, if any, knowledge of the right or wrong way to position patients or of the possible dangers of the use of LTP. Educational efficacy and local implementation have, in this case, gone ahead of medical science.

Our results show that 86% of those provided with training felt confident in the use of LTP, compared to 58% of those who were not provided with training. This indicates the importance of proper training, and we believe training on a regular basis should be implemented in all EMS services. A future study questioning whether the training provided in the use of LTP is sufficient would be useful. Moreover, there are different ways of implementing LTP, and more knowledge on their harms and benefits are required.

We believe that considering the options and all the risks and benefits LTP is a proper way of handling these patients, but further research is a necessity. One of the authors (PKH) is planning further studies to establish the knowledge base and to investigate the actual spinal movement when performing LTP.

## Limitations

Our results should be interpreted with caution. We do not know how large a proportion of the population is served with personnel familiar with LTP. The methodological choice of interviewing supervisors by telephone and personnel by written questionnaires may also have caused a certain bias.

From the ground EMS personnel, we received answers to only 46% of the distributed questionnaires. We believe there most likely is a bias, because those who answered the questionnaire are more likely to be the EMS personnel familiar with LTP. In the unlikely situation that none of the non-responding ground EMS personnel use LTP, the overall percentage of users would be 27% (829/3,025). However, this does not change the conclusion that LTP has been widely implemented, especially when considering the almost complete coverage of the survey with regard to EMS supervisors and air EMS.

## Conclusions

LTP is implemented and used in the majority of the Norwegian EMS, despite little evidence as to its possible benefits and harms. The introduction has to a large extent been followed or preceded by some kind of training and written protocols, and this seems to increase confidence. How the patient is positioned in LTP differs, and the consequence of this is unknown. More research on the utilization and consequences of LTP is essential to establish its evidence base.

## Competing interests

Intellectual interest: PKH developed the LTP method, no economical interest.

The other authors declare that they have no competing interests.

## Authors' contributions

PKH developed the LTP; TW conceived the study; SF, GE, and TW designed the study; SF, GE, and PKH reviewed the literature; SF and GE collected and analyzed the data; and SF, GF, PKH, and TW wrote the manuscript. All of the authors revised and approved the manuscript.
